# An Unusual Case of Pulmonary Cryptococcus

**DOI:** 10.7759/cureus.3707

**Published:** 2018-12-08

**Authors:** Hafiz M Aslam, Kelly A Cann, Kareem H Genena, Syed A Akhtar Trimizi, Mustansir A Mir, Sara L Wallach, Herbert Conaway, Marc Seelagy

**Affiliations:** 1 Internal Medicine, Seton Hall University-Hackensack Meridian School of Medicine, St. Francis Program, Trenton, USA; 2 Internal Medicine, Drexel University College of Medicine, Philadelphia, USA; 3 Internal Medicine, St. Francis Medical Center, Hamilton, USA

**Keywords:** immunocompetent patient, pulmonary involvement, cryptococcosis

## Abstract

Cryptococcal infections are caused by encapsulated fungi Cryptococcus gattii and C. neoformans. Inhalation commonly causes innocuous colonization but may cause meningitis or disseminated disease via hematogenous spread. Cryptococcosis occurs most commonly in immunocompromised patients including those with acquired immunodeficiency syndrome, meningoencephalitis or disseminated disease. However, cryptococcosis can occur as asymptomatic isolated pulmonary nodules in immunocompetent patients. Here we present a unique retrospective case report of a 55-year-old immunocompetent man who presented with pleuritic chest pain, productive cough, dyspnea on exertion, chills, night sweats, and weight loss. A computed tomography scan of his chest revealed multiple ground-glass opacities throughout both lung fields. The results of his autoimmune evaluation and human immunodeficiency virus tests were negative. A biopsy obtained through video-assisted thoracoscopic surgery revealed mucicarmine staining capsules confirming Cryptococcus, requiring treatment with amphotericin, flucytosine, and fluconazole. This case highlights the rarely studied presentation of symptomatic diffuse pulmonary cryptococcal infection in an immunocompetent patient requiring treatment.

## Introduction

Cryptococcal infections are prevalent worldwide (0.4 to 1.3 cases per 100,000 population) and are caused by the fungi Cryptococcus gattii and C. neoformans commonly found in bird droppings, soil, and decaying wood [1]. These infections may primarily involve the lungs, or they may disseminate to other organs. Disseminated cryptococcal infections occur most commonly in immunocompromised patients. However, there are cases of primary pulmonary cryptococcosis in immunocompetent patients. Pulmonary cryptococcosis is contracted by inhaling aerosolized cryptococcal fungi that travel as small basidiospores to the terminal bronchioles and alveoli [2]. In immunocompetent patients, this process typically leads to focal pneumonitis, which is often asymptomatic and can become a latent subclinical infection. Cryptococcosis is often found on examination of biopsies of pulmonary lesions found incidentally on chest imaging. Characteristic pathological findings include small areas of noncalcified granulomatous inflammation of lung parenchyma or perihilar lymph nodes [3,4]. In cases when patients do become symptomatic, they may present with cough, sputum production, hemoptysis, dyspnea, fever, malaise, headache, night sweats or weight loss [5,6]. In immunocompetent hosts with pulmonary cryptococcal infection, an isolated nodule will often resolve spontaneously without antifungal treatment, but more advanced pulmonary disease requires treatment with fluconazole, and disseminated disease requires induction treatment with amphotericin and flucytosine [1,7]. Here, we present a case report of a 55-year-old immunocompetent man who presented with symptomatic, diffuse pulmonary cryptococcosis requiring treatment.

## Case presentation

A 55-year-old intoxicated homeless man presented to the hospital with a cough and chest pain. His past medical history was notable for chronic alcoholism and untreated latent tuberculosis, based on a positive interferon-gamma release assay three years prior to presentation. He had worked as a car mechanic. He smoked half a pack of cigarettes daily and drank a few beers every day. He denied illicit drug use. He reported concern of a cough productive of a moderate amount of yellow sputum that is occasionally streaked with blood. He had the cough for about four weeks with no improvement. His cough was associated with dyspnea on exertion, pleuritic chest pain, chills, night sweats, and a 10-pound weight loss over the past few months.

**Figure 1:**
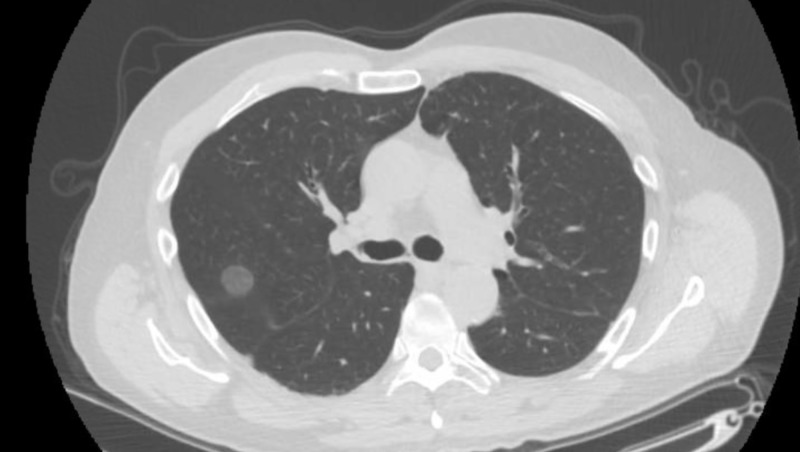
Pulmonary nodule: 18 mm (initial presentation)

**Figure 2:**
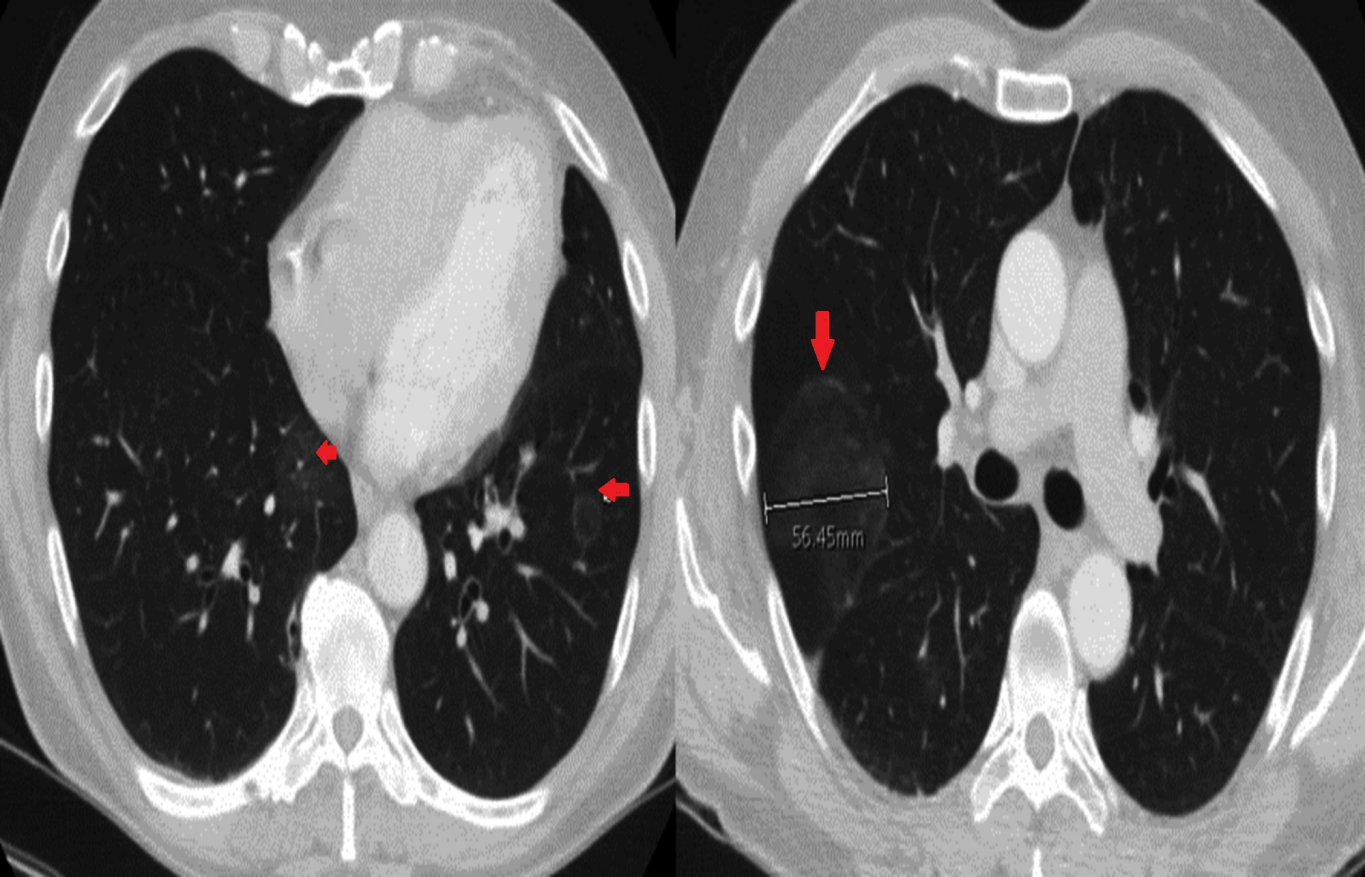
After three months: chest CT showing bilateral ground-glass opacities involving all lung lobes; the largest measured 5.7 cm in diameter CT - computed tomography

The patient’s vital signs revealed a temperature of 98.2°F, a heart rate of 92 beats per minute, a respiratory rate of 18 breaths per minute, and a blood pressure level of 96/55 mmHg. His oxygen saturation was 98% breathing ambient air. The patient’s lung examination revealed normal work of breathing and decreased breath sounds on the left lung fields without wheezing, crackles, or rhonchi. The results of his cardiac, abdominal, and neurological exams were all within the reference range. No lymph nodes were palpated. Results from the laboratory workup included a complete blood count and comprehensive metabolic panel, both of which were within the reference ranges. The findings of his chest radiography were unremarkable. Figure 1 represents the initial presentation of the patient three months ago and Figure 2 represents the computed tomography scan of his chest that revealed bilateral ground-glass opacities involving all lung lobes; the largest measured 5.7 cm in diameter.

The patient was placed in respiratory isolation, and an empiric antibiotic therapy was started to cover community-acquired and aspiration pneumonia. Sputum stain tests for acid-fast bacilli were negative on three consecutive days, and respiratory isolation was discontinued. A Gram stain showed normal respiratory flora. Urine legionella and histoplasma antigen test results remained negative. The results of Streptococcal antigen, Aspergillus antigen, and Fungitell® assays were negative. Human immunodeficiency virus (HIV) serology, anti-nuclear antibody, rheumatoid factor, and antineutrophilic cytoplasmic antibody workup results were negative. The erythrocyte sedimentation rate and complement levels were within reference range. His immunoglobulin panel showed an elevated immunoglobulin E (IgE) level at 3309 IU/mL, a low immunoglobulin G (IgG) level at 601 mg/dL, and a low immunoglobulin M (IgM) level at 25 mg/dL. Immunoglobulin A (IgA) levels were within reference range, and the levels were checked again during follow-up and they were completely normal. Initial decrement in levels might be due to the patient's ongoing illness. The patient underwent a surgical lung biopsy. Pathology testing revealed pulmonary fibrosis, reactive alveolar changes, granulomatous inflammation with focal calcifications, positive fungal stains, a mucicarmine rich capsule, and morphology consistent with cryptococcal infection. There was no evidence of malignancy or vasculitis. On the sixth hospital day, empiric antibiotic therapy with ceftriaxone, azithromycin, and metronidazole was discontinued in favor of treatment specific for cryptococcal pneumonia—14 days of intravenous amphotericin B and oral flucytosine. Following induction, the patient was discharged on oral fluconazole, to continue for six to 12 months. Outpatient follow-up was scheduled with the infectious diseases and immunology clinics. The patient was lost to follow-up after a one-time show-up in the medical clinic.

## Discussion

Cryptococcosis occurs most commonly in immunocompromised patients such as those with an HIV infection, diabetes, chronic liver disease, hematologic malignancies, solid organ or hematopoietic stem cell transplantation, corticosteroid therapy, and those using tumor necrosis factor-alpha antagonists [8]. There are reports of primary pulmonary Cryptococcus infections in immunocompetent patients. Studies in Canada where C. gattii is prevalent reported only 218 cases of cryptococcal infection from 1999–2007, which included only 148 immunocompetent patients, 55.6% of whom required hospitalization for pulmonary symptoms [9]. Our case represents a patient with a rare manifestation of a rare disease—pulmonary cryptococcal infection in an immunocompetent patient. Many immunocompetent patients with pulmonary cryptococcal infection are asymptomatic. In cases when patients do become symptomatic, they typically present, as our patient did, with cough, sputum production, hemoptysis, dyspnea, fever, malaise, night sweats, and weight loss [5,6].

Pulmonary cryptococcal infection is diagnosed based on cultures, antigen testing, and radiographic findings. Cultures obtained from sputum, bronchial lavage, pleural effusion, or tissue biopsies are diagnostic when they grow mucoid cream colonies at 30°C after two to five days [7]. Histology reveals encapsulated yeast with a mucicarmine-positive capsule on light microscopy [7]. These diagnostic histological findings were consistent with the findings from our patient’s video-assisted thoracoscopic surgery (VATS) biopsy, which also revealed encapsulated yeast and a mucicarmine-rich capsule. Cultures can also be obtained from cerebrospinal fluid (CSF) in cases of disseminated disease. Cryptococcal antigen testing is also used for diagnosis of cryptococcal infection. Antigen testing of CSF for immunocompromised patients with meningoencephalitis is reliable with a sensitivity of 93–100 and specificity of 93–98. For a titer of cryptococcal antigen ≥ 1:8, there was 100% sensitivity, 98% specificity, a positive predictive value of 67%, and a negative predictive value of 100%. The measurement of cryptococcal antigen within a bronchoalveolar lavage (BAL) can be a rapid, simple way to diagnose cryptococcal pneumonia in immunosuppressed patients with pneumonia. Cryptococcal antigen testing of BAL and sputum has been inconsistent in the past in immunocompetent patients and is, therefore, not currently used for diagnostic purposes in immunocompetent patients (and the reason the results were negative in our case) [10-11].

Characteristic chest radiographic findings in immunocompetent patients with pulmonary disease include single or multiple discrete, noncalcified pleural nodules. These nodules are often apical and look radiologically similar to cancer, warranting a VATS biopsy as was done in our patient [12]. In immunocompromised patients, chest radiography often shows diffuse pulmonary infiltrates with hilar lymphadenopathy and suggests further disseminated disease [1, 13]. Therefore, our patient's bilateral diffuse ground-glass pulmonary infiltrates without hilar lymphadenopathy and any evidence of disseminated disease make this a unique presentation of isolated pulmonary Cryptococcus.

Treatment for Cryptococcus varies based on disease involvement and host factors. In asymptomatic immunocompetent patients with a single nodule and negative cryptococcal antigen titers, watching and waiting without treatment is acceptable as the nodule often resolves on its own [1]. Immunocompetent patients with mild to moderate pulmonary cryptococcal infection without diffuse infiltrates can be treated with six to 12 months of 400 mg oral fluconazole daily [7]. In patients with meningoencephalitis, disseminated disease, severe pulmonary disease with diffuse infiltrates, or those who are immunocompromised, the treatment is 14 days of induction with flucytosine and amphotericin B, followed by six to 12 months of maintenance fluconazole [7]. Because our patient had pulmonary Cryptococcus with bilateral diffuse infiltrates, he was treated with flucytosine, amphotericin B, and fluconazole.

## Conclusions

The most common forms of immunosuppression other than HIV include glucocorticoid therapy, biologic modifiers, solid organ transplantation, cancer (particularly hematologic malignancy), and conditions such as sarcoidosis and hepatic failure. It is very rare to have a cryptococcal infection in an immunocompetent patient.
